# Risk Factors of Brain Metastasis and Prognosis in HER2-Positive Breast Cancer: A Single-Institution Retrospective Analysis from China

**DOI:** 10.3389/fonc.2022.905065

**Published:** 2022-06-27

**Authors:** Shuang-Long Cai, Zhi-Hong Wang, Xiao-Geng Chen, Lei Han, Guo-Xian Gong, Yan-Ping Chen, Xiu-Quan Lin, Tao Ma, Hong-Dan Chen

**Affiliations:** ^1^ Department of Oncological Surgery, Provincial Clinical Medical College of Fujian Medical University, Fujian Provincial Hospital, Fuzhou, China; ^2^ Department of Hematology, Provincial Clinical Medical College of Fujian Medical University, Fujian Provincial Hospital, Fuzhou, China; ^3^ Department of UItrasonic Diagnosis, Fujian Provincial Hospital, Fuzhou, China; ^4^ Department of Obstetrics and Gynecology, Provincial Clinical Medical College of Fujian Medical University, Fujian Provincial Hospital, Fuzhou, China; ^5^ Department for Chronic and Noncommunicable Disease Control and Prevention, Fujian Provincial Center for Disease Control and Prevention, Fuzhou, China; ^6^ Third Department of Breast Cancer, Tianjin Medical University Cancer Institute and Hospital, National Clinical Research Center for Cancer, Tianjin Key Laboratory of Cancer Prevention and Therapy, Tianjin’s Clinical Research Center for Cancer, Key Laboratory of Breast Cancer Prevention and Therapy, Tianjin Medical University, Ministry of Education, Tianjin, China; ^7^ First Department of Cadre Clinic, Provincial Clinical Medical College of Fujian Medical University, Fujian Provincial Hospital, Fuzhou, China

**Keywords:** brain metastasis, risk factors, prognostic analysis, HER2 positive, breast cancer

## Abstract

**Background:**

Brain metastasis (BM) frequently occurs in HER2-positive breast cancer (BC) patients, but the risk factors of BM in this type of patients are still unknown. Our study aims to assess the risk factors of BM and prognostic analysis in HER2-positive BC patients.

**Methods:**

Univariate analysis used *t*-test, chi-square test, and Fisher’s exact test to find out the risk factors for BM, and multivariable analysis was done with stepwise logistic regression analysis. Prognostic data analysis was estimated by the Kaplan–Meier method.

**Results:**

A total of 228 HER2-positive BC patients were included, of whom 214 patients were postoperative metastatic patients and 14 patients were *de novo* stage IV patients. Through comparing the stratified variables between 51 postoperative metastatic patients with BM and 163 postoperative metastatic patients without BM, the multivariate analysis showed that age ≤40 years (OR 2.321, 95% CI: 1.089 to 4.948) and first metastatic site with lung metastasis (OR 2.168, 95% CI: 1.099 to 4.274) were independent risk factors for BM in HER2-positive BC patients. Prognostic data of all 65 HER2-positive BC patients with BM showed that the time from the diagnosis of BC to the development of breast cancer brain metastasis (BCBM) was 36.3 months (95% CI: 30.0 to 42.1 months). The time from the diagnosis of first recurrence and metastasis stage to the diagnosis of BCBM was 11.35 months (95% CI: 7.1 to 18.4 months). The time from the diagnosis of BCBM to the time of follow-up was 24.1 months (95% CI: 13.9 to 37.5 months). Up until the time of follow-up data, a total of 38 patients had died, and the time from the diagnosis of BM of these 38 patients to death was 11.0 months (95% CI: 9.0 to 20.4 months).

**Conclusion:**

The prognosis of HER2-positive BC patients with BM was poor due to the lack of effective treatments for BM. Age ≤40 years and first metastatic site with lung metastasis were the independent risk factors for BM in HER2-positive BC patients. Future research about pre-emptive medical interventions may help to improve the prognosis of HER2-positive BC patients with high risk to develop BM.

## Introduction

The latest Global Cancer Statistics 2020 shows that breast cancer (BC) has become the malignant tumor with the highest morbidity and the second highest mortality (1). The major mortality reason of BC is the recurrence and metastasis of distant organs. Normally, the progression of disease for most BC patients is slow. Brain metastasis (BM) is preceded by metastases to other organs like lung, liver, or bone. The incidence of clinically evident BM among stage IV BC patients is estimated to be 10% to 16%. These figures underestimate the true incidence, given that BMs are found in 36% of patients at autopsy (2–4). Since most antitumor drugs do not cross the blood–brain barrier, to exert their antitumor properties, the brain has become an important sanctuary site for tumor cells. Therefore, the clinical treatment of BM in BC patient is a difficult problem. Relevant data show that the median survival time of BCBM (breast cancer brain metastasis) patients is less than 6 months, and only 20%–40% of patients survive longer than 1 year (5).

Besides triple-negative BC, HER2-positive BC, which accounted for approximately 10%–15% of BC, has the highest incidence of BM (6, 7). With the development of new drugs and the continuous improvement of drug therapy, the systematic treatment of HER2-positive advanced BC patients has been greatly improved in recent years. Theoretically, better systemic treatment would lead to better control of disease. However, an increasing proportion of patients have been observed to be suffering from BM often at a time when their extracranial disease is apparently under control in clinical practice (8, 9). The identification of HER2-positive BC patients with high risk factors to develop BM would enable pre-emptive intervention such as prophylactic treatment or diagnostic screening with the potential to improve the prognosis of these patients. Reported risk factors for BM in BC patients include young age at first diagnosis, presence of lung metastases, short disease-free survival, hormone receptor (HR)-negative tumors, triple-negative tumor subtype, HER2 overexpression, and BRCA1 phenotype (2, 8, 10–15). However, most of the studies included unselected patients with BC, whereas less is known about risk factors for BM for cohorts of only HER2-positive BC. In this retrospective, single-institution analysis, we aim to identify the high-risk factors for HER2-positive BC patients who would be more likely to progress to BM. Maybe these patients may benefit from the pre-emptive medical intervention in the future. Moreover, we assess the prognostic survival of HER2-positive BC patients with BM in our study.

## Materials and Methods

### Patients

This retrospective analysis included 228 consecutive HER2-positive (immunohistochemistry 3+ or FISH-positive) pathologically confirmed BC patients treated in Fujian Provincial Hospital from January 2004 to January 2021. Male patients, HR+HER2- patients, triple-negative patients, patients with a history of other malignant tumors, or *de novo* metastatic HR+HER2+ and HR-HER2+ patients without BM were excluded.

Follow-up data were gathered until December 31, 2021. HER2 status was determined with immunohistochemistry (IHC) and *in situ* hybridization at the time of the first biopsy or breast surgery and classified according to the American Society of Clinical Oncology/College of American Pathologists clinical practice guidelines for HER2 testing of 2007 and 2013, respectively, and the Belgian Guidelines for HER2 testing (16, 17). HR status was determined by IHC using the Allred scoring system (18). Metastatic lesions were grouped into the following categories: Lung, bone, liver, brain, chest wall or regional lymph nodes metastasis.

Our follow-up treatment strategy is as follows: routine breast color ultrasound, liver color ultrasound, ECT, x-ray, chest, and cranium CT plain scan for every initial treatment patient to exclude the possibility of distant metastasis. In the first 3 years after surgery, we regularly reviewed breast color ultrasound, liver color ultrasound, x-ray, and chest CT plain scan every 3 months. Patients who survived 3–5 years after surgery were regularly reviewed with these examinations every 6 months. Patients who survived more than 5 years after surgery were regularly reviewed with these examinations every 1 year. ECT and cranial examination are not routine review items for postoperative patients, except when the patient has symptoms associated with bone metastasis or BM. Once distant metastasis was confirmed in follow-up patients, liver, lung, cranium CT, and ECT examinations were routinely performed at the same time, and MRI examinations were performed to help determine whether the patients were accompanied by multiple organ metastasis if necessary. Every advanced BC patient was evaluated by CT examination every 2 months. Patients with stable BMs underwent cranium CT/MRI examination every 2–3 months. BM patients with progression disease were evaluated with cranium CT/MRI examination every month if necessary.

### Statistical Analysis

Statistical analyses were conducted using SPSS version 22.0. Univariate analysis used *t*-test, chi-square test, and Fisher’s exact test. *t*-test was done for comparison of quantitative indicators between 2 groups, and chi-square test and Fisher’s exact test were used for comparison of sample rates between 2 groups. Multivariate analysis used stepwise logistic regression, and all variables in the univariate analysis with a *p*-value <0.1 were included in multivariate analysis. The Kaplan–Meier method was used to estimate the time from metastatic disease diagnosis until the development of BM, and was also used to estimate the time from the development of BM to death or follow-up data.

## Results

**Figure 1:**
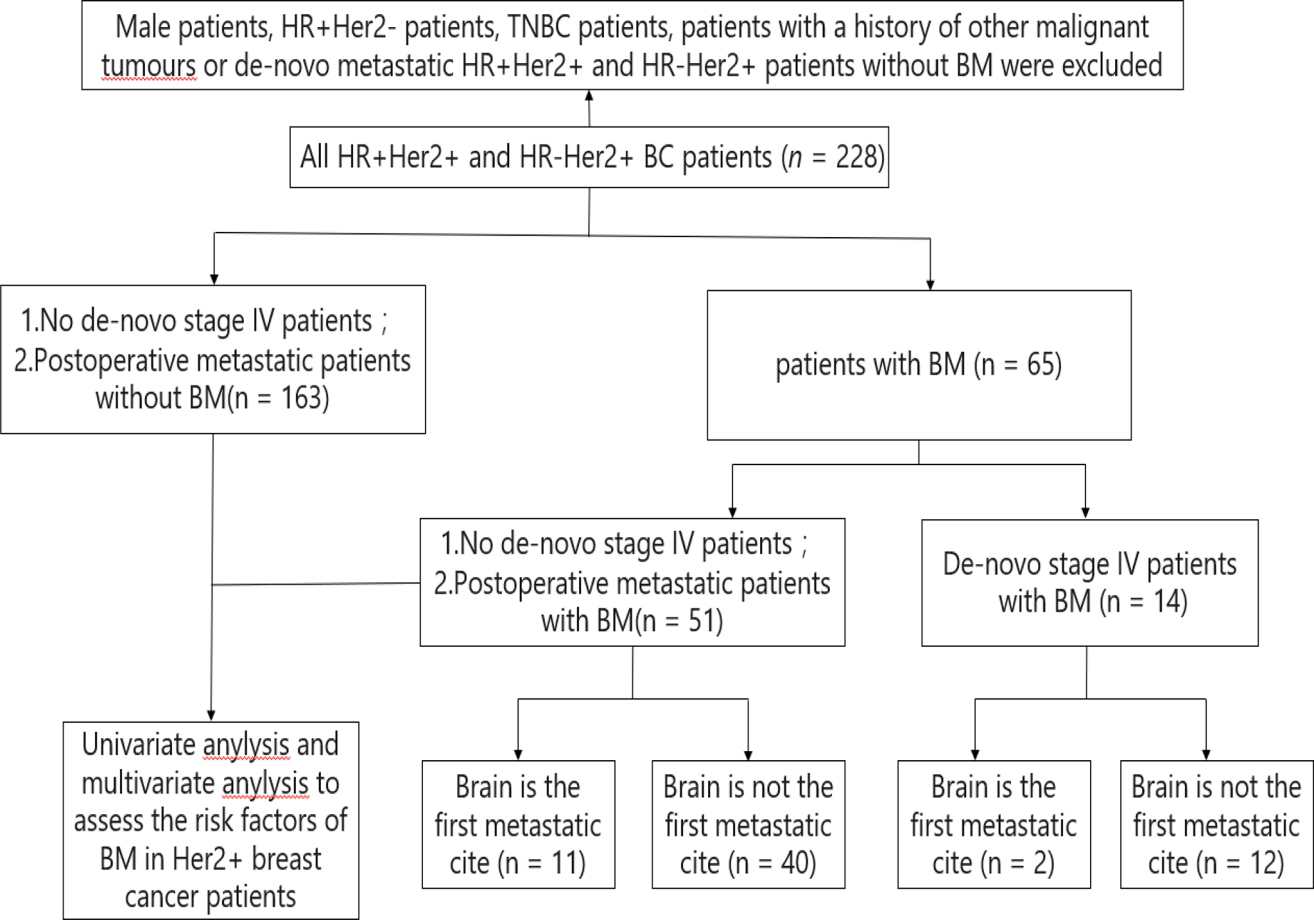
Constitution of the study population. BC, breast cancer; BM, brain metastasis; HR, hormone receptor; HR+, HR positive; HR-, HR negative; Her2, human epidermal growth factor receptor 2; Her2+, Her2 positive; Her2-, Her2 negative; TNBC, triple negative breast cancer.

BM occurred in 65 of the 228 enrolled HER2-positive BC patients. The other 163 patients are postoperative metastatic patients without BM. These 163 patients are not *de novo* stage IV patients. Among the 65 patients with BM, 14 of them were *de novo* stage IV patients with BM, and the other 51 patients are postoperative metastatic patients with BM (**Figure 1**). Among the 65 patients, 13 patients had BM as the first metastasis cite. Up to the follow-up data (December 31, 2021), 38 patients died, and 37 patients were still in follow-up.

Comparing the stratified variables between 51 postoperative metastatic patients with BM and 163 postoperative metastatic patients without BM, the result of univariate analysis showed that age ≤ 40 years and first metastatic site with lung metastasis were significantly associated with the risk of BM in patients with HER2-positive BC (**Table 1**). Variables with *p* < 0.1 in univariate analysis were included in multivariate analysis, and it was found that age ≤40 years and first metastatic site with lung metastasis were independent risk factors for BM in HER2-positive BC patients (**Table 2**).

**Table 1:** Risk factors of brain metastasis in Her2 positive postoperative breast cancer patients according to stratified variables: univariate analysis.

Postoperative metastatic Postoperative metastatic			
Variables	Patients without BM (n=163)	Patients with BM (n=51)	P value
	No. (%)	No. (%)	
Age at dx (years)	49.09±10.09	45.55±10.09	0.03
Age at diagnosis			0.042
≤40 years	32 (19.63)	17 (33.33)	
>40 years	131 (80.37)	34 (66.67)	
Menopausal status at diagnosis			0.077
Premenopausal	89 (54.60)	35 (68.63)	
Postmenopausal	74 (45.40)	16 (31.37)	
Family history			0.107
No	156 (95.71)	45 (88.24)	
Yes	7 (4.29)	6 (11.76)	
Type of surgery			0.616
Radical surgery	158 (96.93)	48 (94.12)	
Breast conserving surgery	5 (3.07)	3 (5.88)	
Pathology type			0.535
Invasive ductal carcinoma	122 (74.85)	36 (70.59)	
Mixt	38 (23.31)	15 (29.41)	
Others	3 (1.84)	0 (0.00)	
Tumour grade			0.471
G1	3 (1.84)	0 (0.00)	
G2	102 (62.58)	27 (52.94)	
G3	52 (31.90)	22 (43.14)	
Missing	6 (3.68)	2 (3.92)	
Tumour size staging			0.322
pT1	48 (29.45)	15 (29.41)	
pT2	92 (56.44)	24 (47.06)	
pT3-4	19 (11.66)	11 (21.57)	
Missing	4 (2.45)	1 (1.96)	
Nodal staging			0.221
pN0	47 (28.83)	21 (41.18)	
pN1	47 (28.83)	8 (15.69)	
pN2	30 (18.40)	11 (21.57)	
pN3	37 (22.70)	10 (19.61)	
Missing	2 (1.23)	1 (1.96)	
Lymph nodes metastatic status			0.091
No metastasis	47 (29.19)	21 (42.00)	
Metastasis	114 (70.81)	29 (58.00)	
Estrogen receptor status			0.082
Negative	70 (42.94)	29 (56.86)	29 (56.86)
Positive	93 (57.06)	22 (43.14)	
Progesterone receptor status			0.069
Negative	85 (52.15)	34 (66.67)	
Positive	78 (47.85)	17 (33.33)	
Time to first distant relapse			0.139
≤2 years	64 (39.26)	26 (50.98)	
>2 years	99 (60.74)	25 (49.02)	
Type of chemotherapy			0.496
Anthracyclines	9 (5.52)	4 (7.84)	
Taxanes	12 (7.36)	5 (9.80)	
Anthracyclines+taxanes	138 (84.66)	40 (78.43)	
Other	2 (1.23)	0 (0.00)	
None	2 (1.23)	2 (3.92)	
Type of anti-HER2 treatment			0.315
Trastuzumab	66 (40.49)		
Trastuzumab+pertuzumab	0 (0.00)	1(1.96)	
None	97(59.51)	30 (58.82)	
Adjuvant endocrine therapy			0.248
No	84 (51.53)	31 (60.78)	
Yes	79 (48.47)	20 (39.22)	
Adjuvant radiotherapy			0.439
No	90 (55.21)	25 (49.02)	
Yes	73 (44.79)	26 (50.98)	
First metastatic site			
Lung metastasis			0.027
No	105 (64.42)	24 (47.06)	
Yes	58 (35.58)	27 (52.94)	
Liver metastasis			
No	105 (64.42)	24 (47.06)	
Yes	58 (35.58)	27 (52.94)	
Bone metastasis			0.135
No	120 (73.62)	32 (62.75)	
Yes	43 (26.38)	19 (37.25)	
Metastatic cite in chest wall or regional lymph nodes			0.407
No	66 (40.49)	24 (47.06)	
Yes	97 (59.51)	27 (52.94)	

BM, brain metastasis dx, diagnosis; Family history,HBOC related cancer history.

**Table 2:** Multivariate Analysis for risk factors of brain metastases in Her2 positive postoperative breast cancer patients.

Variable	Estimate	Se	z	Wald	p	OR(95%CI)
Age at diagnosis						
>40 years	ref					
≤40 years	0.842	0.386	2.181	4.756	0.029	2.321 (1.089, 4.948)
Lymph nodes metastatic status						
No metastasis	ref					
Metastasis	-0.586	0.349	-1.677	2.813	0.094	0.557 (0.281, 1.104)
Estrogen receptor status						
Negative	ref					
Positive	-0.138	0.532	-0.259	0.067	0.795	0.871 (0.307, 2.472)
Progesterone receptor status						
Negative	ref					
Positive	-0.668	0.555	-1.203	1.447	0.229	0.513 (0.173, 1.523)
First metastatic site						
Lung metastasis						
No	ref					
Yes	0.774	0.346	2.234	4.989	0.026	2.168 (1.099, 4.274)

All variables in the univariate analysis with a P value <0.1 were included in multivariate analysis.

**Figure 2:**
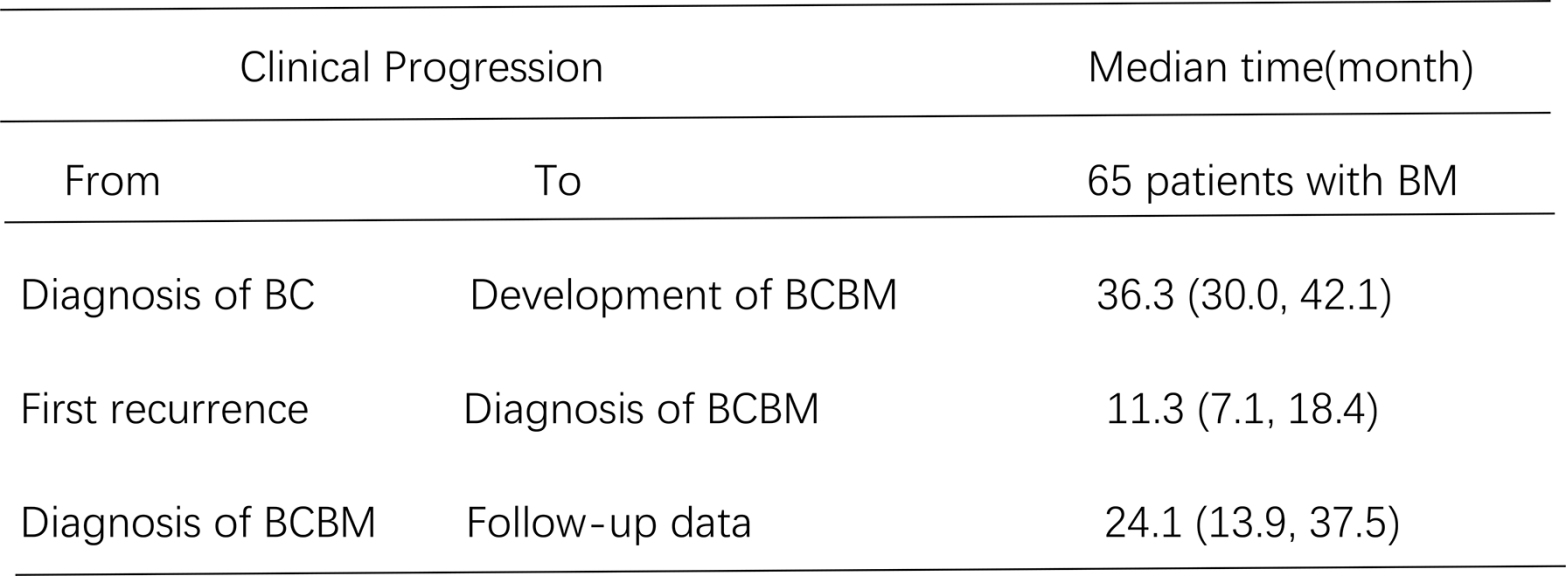
Different clinical progression of median time for 65 Her 2 positive breast cancer patients with BM.

Local treatments after BMs in 65 patients were as follows: 38 patients received only craniocerebral radiotherapy, 9 patients received only surgical resection of BM, 5 patients received craniocerebral radiotherapy combined with surgical resection of BMs, and 13 patients did not receive local treatment. Medical treatment after BMs in 65 patients were as follows: 5 patients had no medical treatment, and 60 patients had medical treatment (11 of them had no local treatment and only received medical treatment). Prognostic data analysis was estimated by the Kaplan–Meier method. Among all 65 HER2-positive BC patients with BM, the time from the diagnosis of BC to the development of BCBM was 36.3 months (95% CI: 30.0 to 42.1 months). The time from the diagnosis of first recurrence and metastasis stage to diagnosis of BCBM was 11.3 months (95% CI: 7.1 to 18.4 months). The time from the diagnosis of BCBM the time of follow-up was 24.1 months (95% CI: 13.9 to 37.5 months) (**Figure 2**). Up until the time of follow-up, 38 patients died, and the time of these 38 patients from the diagnosis of BM to death was 11.0 months (95% CI: 9.0 to 20.4 months).

## Discussion

Over the past decades, the diagnosis and treatment of BC patients have achieved remarkable improvements. Due to the lack of effective treatments, BM is still associated with poor results (19–21). In this study, we described the prognostic data of 65 HER2-positive BC patients with BM. The time from the diagnosis of BC to the development of BCBM was 36.3 months (95% CI: 30.0 to 42.1 months). The time from the diagnosis of first recurrence and metastasis stage to diagnosis of BCBM was 11.3 months (95% CI: 7.1 to 18.4 months). The time from the diagnosis of BCBM to the time of follow-up was 24.1 months (95% CI: 13.9 to 37.5 months). Up until the time of follow-up, the time of the 38 dead patients from the diagnosis of BM to death was 11.0 months (95% CI: 9.0 to 20.4 months). Based on these survival data that we obtained in this study, we have to say that HER2-positive BC patients remain at a continuous risk of brain relapse for a prolonged period of time after diagnosis of BC and diagnosis of recurrence and metastasis stage. Therefore, the prevention and management of BM still pose unique clinical challenges. Despite the short overall survival after the diagnosis of BCBM, the results in our study seem to be better compared to those reported previously in unselected series of BCBM patients (5, 22–24). This may be related to earlier diagnosis of HER-positive BC, and better standardized systematic treatment of HER-positive BC, particularly the use of trastuzumab, pertuzumab, and small-molecule tyrosine kinase inhibitors. Furthermore, more effective management of BM and more humane supportive treatment also play an important role.

In this study, we also included a series of HER2-positive postoperative BC patients to assess the risk factors of BM. We included the clinical and pathological factors in predicting risk factors of BM in HER2-positive BC patients. Among these clinicopathological indicators, primary tumor size and lymph node metastatic status are the important indicators to reflect the characteristics of BC. Traditional perspectives consider the idea that BC patients with larger tumors and more lymph node metastases are more likely to have recurrence and metastasis. Some scholars’ research has found that tumor size >2 cm and axillary lymph nodal involvement were the risk factors for the development of BM in HER2-positive BC patients (25). However, we did not find this statistical significance in our study.

Besides the primary tumor size and lymph node metastatic status, HR status and tumor grade could also reflect the characteristics of BC. Related literature reported that HR status and tumor grade were considered an adverse prognostic factor in unselected BC patients, and also increased the risk of BM (26–28). Knowledge on the predictive value of HR status and tumor grade for BM in HER2-positive BC patients is scarce. However, we also did not find any association between these two factors and the risk of BM in our study. Additionally, univariate and multivariate analyses show that no statistical significance has been found among menopausal status at diagnosis, family history, type of surgery and pathological type, probably due to the small number of patients enrolled in our study and the selectivity bias in our retrospective study. However, age ≤40 years (OR 2.321, 95% CI 1.089 to 4.948) did increase the risk of BM in HER2-positive BC patients. The possible reason is that young patients may display an increased propensity for BM due to the longer expected survival.

At present, no positive correlation has been found between different postoperative treatments and the occurrence of BM in HER2-positive BC patients due to lack sufficient studies. This is consistent with the results of our study. As for the postoperative anti-HER2 targeted therapy, two meta-analyses reported an increased incidence of BM (29, 30). This may extend the survival time to such a degree as to display an increased propensity for BM due to a better control of extracranial disease with trastuzumab (5, 8). However, our study did not find that anti-HER2-targeted therapy was associated with the occurrence of BM.

In our study, we also analyzed the relationship between the relevant metastatic variables and BM in HER2-positive BC patients. Variables associated with the recurrence and metastasis included the time from initial diagnosis to distant relapse, lung metastasis as the first metastatic event, liver metastasis as the first metastatic event, bone metastasis as the first metastatic event, and chest wall or regional lymph node metastasis as the first metastatic event. Different from the Renata Duchnowska’s study report, the time from initial diagnosis to distant relapse shorter than 2 years did not increase the risk of BM in our study. Similarly, liver metastasis as the first metastatic event, bone metastasis as the first metastatic event, and chest wall or regional lymph node metastasis as the first metastatic event also had no significant statistical significance. However, univariate and multivariate analysis showed that lung metastasis as the first metastatic event (OR 2.168, 95% CI: 1.099 to 4.274) was the independent risk factor for BM in HER2-positive BC patients in our study. The possible reasons are as follows: (1) due to the fact that patients with liver metastasis generally have a more serious disease and fast progress, they may have died before the occurrence of BM; (2) due to the slow progress and insufficient follow-up time, BM was not found in patients with bone metastasis and chest wall or regional lymph node metastasis; and (3) correlative studies have reported that the expression of the epidermal growth factor receptor ligand and the cyclooxygenase COX2 is associated with brain and lung metastasis of BC, but not associated with liver and bone metastasis (31, 32). In this study, lung metastasis as the first metastatic event is positively correlated with BM, which may be related to the above molecular mechanism.

## Limitation

There are several limitations that need to be considered in our study: (1) This is a retrospective study from a single center, which may result in selective bias. (2) The patients enrolled in our study conveys a time period of 17 years. During these years, the postoperative diagnosis and treatments of HER2-positive BC patients have changed, and the management of BM has also changed. Even in the same medical center, patients’ final medical options are different, because they depended on different medical teams’ treatment plans, disease and economic factors of patients, and the availability and tolerance of drugs. Therefore, the potential bias caused by heterogeneity of patient populations and inconsistent therapeutic approaches cannot also be ignored. (3) The total number of HER2-positive BC patients with or without BM in this study are still not large enough. We need to expand our sample size to analyze the risk factors of BMs and prognosis of HER2-positive BC patients in the future. (4) The follow-up time is not long enough in this study. Until the follow-up time, nearly half of the patients are still being followed up, and we will continue to follow up so that we could obtain more information about the prognosis of HER2-positive BC with BM in the future.

## Conclusion

Combined with the findings in our single-center study, we demonstrate that HER2-positive BC patients with BM have a poor prognosis. Risk factors for BM in HER2-positive BC patients are age ≤40 years and first metastatic site with lung metastasis. Considering the limitations in our study, we have the following considerations: (1) We expect that multi-center and large sample size studies could continue to explore the risk factors of BM and prognosis in HER2-positive BC in the future, so that we could establish a prediction model to predict the likelihood of BM in HER2-positive BC. Moreover, we expect that correlational researchers will be able to explore the corresponding predictive markers to predict the BM in HER2-positive BC with the help of genetic detection in the future. Through these efforts, we can screen out the real high-risk population with BM in HER2-positive BC patients. It would provide important references for our clinical decision-making. (2) In recent years, relevant studies have reported that small-molecule tyrosine kinase inhibitors could improve the progression and prognosis of BM in HER2-positive BC patients (33–35). We expect that there could be some clinical trials about pre-emptive medical interventions in the future. For example, randomized controlled trials are conducted in metastatic HER2-positive BC patients who had high risk factors to develop BM to explore whether the regular head MRI screening would be good for the early diagnosis and treatment, or there could be clinical trials to explore whether small-molecule tyrosine kinase inhibitors used in adjuvant therapy or first-line advanced salvage treatment would decrease the occurrence of BM, and ultimately improve the prognosis of brain metastasis in HER2-positive BC patients.

## Data Availability Statement

The datasets presented in this study can be found in online repositories. The names of the repository/repositories and accession number(s) can be found in the article/supplementary material.

## Ethics Statement

This study was approved by the Ethics Committee of Fujian Provincial Hospital. Written informed consent was obtained from all patients included in the study.

## Author Contributions

S-LC and Z-HW contributed to the conception and design of the study. S-LC, Z-HW, X-GC, LH, G-XG and X-QL developed the methodology. X-GC, Y-PC, X-QL, G-XG, and MT took part in the acquisition, analysis, and interpretation of the data. S-LC, Z-HW, X-GC and H-DC ,wrote, reviewed, and/or revised the manuscript. LH, X-GC and H-DC supervised the study. All authors contributed to the article and approved the submitted version.

## Funding

This work was supported by joint funds for the innovation of science and technology, Fujian province (2020Y9027).

## Conflict of Interest

The authors declare that the research was conducted in the absence of any commercial or financial relationships that could be construed as a potential conflict of interest.

## Publisher’s Note

All claims expressed in this article are solely those of the authors and do not necessarily represent those of their affiliated organizations, or those of the publisher, the editors and the reviewers. Any product that may be evaluated in this article, or claim that may be made by its manufacturer, is not guaranteed or endorsed by the publisher.

## Abbreviations

BM brain metastasis; BC breast cancer; BCBM breast cancer brain metastasis; HR hormone receptor; HR+ HR positive; HR- HR negative; HER2 human epidermal growth factor receptor 2; HER2+ HER2 positive; HER2- HER2 negative; TNBC triple-negative breast cancer.
