# Erratum: Risk factors of brain metastasis and prognosis in HER2-positive breast cancer: A single-institution retrospective analysis from China

**DOI:** 10.3389/fonc.2022.1027199

**Published:** 2022-09-20

**Affiliations:** Frontiers Media SA, Lausanne, Switzerland

**Keywords:** brain metastasis, risk factors, prognostic analysis, HER2 positive, breast cancer

Due to a production error, the affiliation linked to Yan-Ping Chen and Hong-Dan Chen were incorrect. The correct author affiliations linked to the authors should be Shuang-Long Cai1†, Zhi-Hong Wang2†, Xiao-Geng Chen1, Lei Han1, Guo-Xian Gong3,Yan-Ping Chen4, Xiu-Quan Lin5, Ma Tao6, and Hong-Dan Chen7*.

There was also an error in **Table 1**. The heading “Postoperative metastatic patients without BM (n=163)” should be “Postoperative metastatic patients with BM (n=51)”.

**Table 1:** Risk factors of brain metastasis in Her2 positive postoperative breast cancer patients according to stratified variables: univariate analysis.

Postoperative metastatic Postoperative metastatic			
Variables	Patients without BM	Patients with BM	P value
	(n=163)	(n=51)	
	No. (%)	No. (%)	
Age at dx (years)	49.09 ± 10.09	45.55 ± 10.09	0.03
Age at diagnosis			0.042
≤40 years	32 (19.63)	17 (33.33)	
>40 years	131 (80.37)	34 (66.67)	
Menopausal status at diagnosis			0.077
Premenopausal	89 (54.60)	35 (68.63)	
Postmenopausal	74 (45.40)	16 (31.37)	
Family history			0.107
No	156 (95.71)	45 (88.24)	
Yes	7 (4.29)	6 (11.76)	
Type of surgery			0.616
Radical surgery	158 (96.93)	48 (94.12)	
Breast conserving surgery	5 (3.07)	3 (5.88)	
Pathology type			0.535
Invasive ductal carcinoma	122 (74.85)	36 (70.59)	
Mixt	38 (23.31)	15 (29.41)	
Others	3 (1.84)	0 (0.00)	
Tumour grade			0.471
G1	3 (1.84)	0 (0.00)	
G2	102 (62.58)	27 (52.94)	
G3	52 (31.90)	22 (43.14)	
Missing	6 (3.68)	2 (3.92)	
Tumour size staging			0.322
pT1	48 (29.45)	15 (29.41)	
pT2	92 (56.44)	24 (47.06)	
pT3-4	19 (11.66)	11 (21.57)	
Missing	4 (2.45)	1 (1.96)	
Nodal staging			0.221
pN0	47 (28.83)	21 (41.18)	
pN1	47 (28.83)	8 (15.69)	
pN2	30 (18.40)	11 (21.57)	
pN3	37 (22.70)	10 (19.61)	
Missing	2 (1.23)	1 (1.96)	
Lymph nodes metastatic status			0.091
No metastasis	47 (29.19)	21 (42.00)	
Metastasis	114 (70.81)	29 (58.00)	
Estrogen receptor status			0.082
Negative	70 (42.94)	29 (56.86)	
Positive	93 (57.06)	22 (43.14)	
Progesterone receptor status			0.069
Negative	85 (52.15)	34 (66.67)	
Positive	78 (47.85)	17 (33.33)	
Time to first distant relapse			0.139
≤2 years	64 (39.26)	26 (50.98)	
>2 years	99 (60.74)	25 (49.02)	
Type of chemotherapy			0.496
Anthracyclines	9 (5.52)	4 (7.84)	
Taxanes	12 (7.36)	5 (9.80)	
Anthracyclines+taxanes	138 (84.66)	40 (78.43)	
Other	2 (1.23)	0 (0.00)	
None	2 (1.23)	2 (3.92)	
Type of anti-HER2 treatment			0.315
Trastuzumab	66 (40.49)	20(39.22)	
Trastuzumab+pertuzumab	0 (0.00)	1(1.96)	
None	97(59.51)	30 (58.82)	
Adjuvant endocrine therapy			0.248
No	84 (51.53)	31 (60.78)	
Yes	79 (48.47)	20 (39.22)	
Adjuvant radiotherapy			0.439
No	90 (55.21)	25 (49.02)	
Yes	73 (44.79)	26 (50.98)	
First metastatic site			
Lung metastasis			0.027
No	105 (64.42)	24 (47.06)	
Yes	58 (35.58)	27 (52.94)	
Liver metastasis			0.429
No	127 (77.91)	37 (72.55)	
Yes	36 (22.09)	14 (27.45)	
Bone metastasis			0.135
No	120 (73.62)	32 (62.75)	
Yes	43 (26.38)	19 (37.25)	
Metastatic cite in chest wall or regional lymph nodes			0.407
No	66 (40.49)	24 (47.06)	
Yes	97 (59.51)	27 (52.94)	

BM, brain metastasis dx, diagnosis; Family history, HBOC related cancer history.

The corrected **Table 1** and its caption “**Table 1** Risk factors of brain metastasis in Her2 positive postoperative breast cancer patients according to stratified variables: univariate analysis” appear below.

The publisher apologizes for these mistakes. The original version of this article has been updated.

